# The Dutch-translated cultural competence assessment scale for nurses: Cross-cultural adaptation and validation

**DOI:** 10.1016/j.ijnsa.2025.100303

**Published:** 2025-01-29

**Authors:** P.B. Sutharson, J.M. Maaskant, A.M. Eskes

**Affiliations:** a Elkerliek hospital, Department of Cardiology, Wesselmanlaan 25, Helmond, the Netherlands; b Amsterdam UMC location University of Amsterdam, Epidemiology and Data Science, Meibergdreef 9, Amsterdam, the Netherlands; c Amsterdam UMC location University of Amsterdam, Emma Children's Hospital, Meibergdreef 9, Amsterdam, the Netherlands; d Amsterdam UMC location University of Amsterdam, Department of Internal Medicine, Meibergdreef 9, Amsterdam, the Netherlands; e Amsterdam UMC location University of Amsterdam, Department of Surgery, Meibergdreef 9, Amsterdam, the Netherlands; f Menzies Health Institute Queensland and School of Nursing and Midwifery, Griffith University, Gold Coast, G01 2.03 Gold Coast campus Griffith University, QLD 4222, Australia; g Faculty of Health, Center of Expertise Urban Vitality, Amsterdam University of Applied Sciences, Amsterdam, the Netherlands

**Keywords:** Nursing, Cultural competency, Dutch, Nurses, Psychometrics

## Abstract

**Background:**

Researchers have revealed significant disparities in safety events and patient outcomes between minority and non-minority populations, highlighting the need for a deeper understanding of cultural competence. As frontline caregivers, nurses play a key role in providing culturally-sensitive care. Measuring cultural competence can be challenging. The Cultural Competence Assessment, developed and validated in the United States of America, can help to address these challenges.

**Method:**

The aim was to determine the translation and cross-cultural adaptation, while evaluating the psychometric properties, of the Dutch version of the Cultural Competence Assessment among Dutch nurses. We used a cross-sectional design and conducted the study in two phases. Nurses from all over the Netherlands were recruited through the newsletter of the Dutch nurses association and internal distribution by healthcare organisations. The first phase focused on cross-cultural adaptation and translation. The second phase focused on psychometric testing. Data were analysed using Confirmatory Factor Analysis (CFA). Internal consistency was evaluated, utilising McDonald's omega

**Results:**

The sample consisted of 447 Dutch nurses working in different healthcare settings. The original Cultural Competence Assessment consisting of 29 items was reduced to 27 items in the first phase of the study. After CFA, the best fit was obtained with a two-factor model consisting of 15 items, with a proficient level of internal consistency.

**Conclusion:**

With the two-factor, 15-item Dutch Cultural Competence Assessment, we have provided Dutch nurses with an instrument to self-assess their cultural competence, paying special attention to their awareness, sensitivity, and behaviour when caring for people with cultural differences

**What is already known**

• Cultural competence is a key competency for nurses to provide effective care to diverse patient populations.
• Measuring cultural competence can be challenging, particularly in the Netherlands where there are currently no validated Dutch measuring tools.

Alt-text: Unlabelled box

**What this paper adds**

• With the now-validated and cross-culturally adapted Dutch Cultural Competence Assessment, Dutch nurses can self-assess their cultural competence.
• The Dutch Cultural Competence Assessment can help nurses to consider their cultural awareness, sensitivity and behaviour while interacting with others.
• The Dutch Cultural Competence Assessment contributes to the provision of culturally-sensitive and compassionate care in the Netherlands

Alt-text: Unlabelled box

## 1 Introduction

In the Netherlands, where more than 26 % of the population has a minority background (CBS, 2023b), the provision of culturally sensitive healthcare has become an essential aspect of ensuring high-quality and equitable care for all individuals (Health Policy Institute, 2024). However, researchers have revealed significant disparities in safety events and patient outcomes between minority and non-minority populations (Institute of Medicine (US), 2003; Sze et al., 2015; Chauhan et al., 2020). These disparities highlight the need for a deeper understanding of cultural competence.

Given that 60 % of registered healthcare workers in the Netherland are nurses (CBS, 2017), they play a key role as frontline caregivers in providing culturally-sensitive care that respects diverse backgrounds and values. Studies have suggested that culturally sensitive nursing care results in more effective communication, higher patient satisfaction, and better adherence to treatment (Josipovic, 2000; Beach et al., 2005). By embracing and recognising their cultural competence, healthcare providers can foster trust, establish meaningful communication, and deliver personalised care that is sensitive to the diverse needs and preferences of patients (CVIMS, 2024).

Cultural competence in nursing is defined as a continuous and dynamic process through which nurses develop the skills, knowledge, and attitudes necessary to deliver care that is not only safe, effective, and of high quality, but also specifically tailored to the cultural backgrounds, values, beliefs, and needs of individuals from diverse populations. The fundamental elements of cultural competence can be classified into four core categories: awareness, knowledge, sensitivity, and skills (Sharifi, Adib-Hajbaghery and Najafi, 2019). The concept of "culture" includes more than just factors like ethnicity, race, and ancestry. It also extends to shared customs and beliefs among groups sharing common traits, such as individuals with disabilities, sexual and gender minorities, people from diverse socio-economic backgrounds, and members of various religious or spiritual groups (Lin, 2020).

Nurses can use measurement tools to help them consider their cultural skills, awareness, and knowledge while interacting with others and recognise what they can do to become more effective when working in diverse environments. Organisations can use such tools to evaluate and address systematically cultural competence gaps, which occur when healthcare providers are not fully equipped to meet the needs of diverse patients due to cultural differences, such as cultural beliefs, values, or traditions, within their nursing workforce. One clear example of how nurses' personal perspectives may influence direct patient care is in the context of assisted dying. Although it is legalised in many countries, culture plays a significant role in shaping views on this issue. However, it is important to recognise that culture is not static, and individuals within the same cultural group can hold diverse perspectives on the same topic (Bloomer et al., 2024).

Until now, measuring cultural competence has been challenging (Kumas-Tan et al., 2007). Several measurement tools have been developed to explore the nurses’ ability to provide culturally-sensitive care, each with varying focuses and approaches (Gozu et al., 2007; Loftin et al., 2013; Jager et al., 2021). One of the measurement tools, the self-reported Cultural Competence Assessment scale, has been found to be a reliable and valid instrument for the assessment of cultural competence in healthcare (Doorenbos et al., 2005). This self-assessment tool is designed to help healthcare professionals explore their individual cultural competence, and to identify areas of strength and opportunities for ongoing professional development. The original Cultural Competence Assessment scale was developed and validated in the United States of America and has since been psychometrically evaluated in several languages, including German and Korean (Chae et al., 2018; Osmancevic et al., 2022). However, a validated version of the Cultural Competence Assessment to measure cultural competence among Dutch nurses is not yet available. In this study, we aimed to establish the translation and cross-cultural adaptation, while evaluating the validity and reliability of the Dutch version of the Cultural Competence Assessment among Dutch nurses.

## 2 Method

### 2.1 Instrument

We used the Cultural Competence Assessment scale, developed by Doorenbos et al. The Cultural Competence Assessment is a self-reported questionnaire that has been psychometrically evaluated among healthcare professionals (Doorenbos et al., 2005). The decision to utilise the Cultural Competence Assessment was based on its strong psychometric properties. Additionally, its particular emphasis on behaviour, awareness, and sensitivity distinguishes it from other comparable instruments, as it captures both the attitudinal and behavioural aspects of cultural competence. The Cultural Competence Assessment is organised into two subscales: (1) awareness and sensitivity, and (2) behaviour. The subscale measuring awareness and sensitivity assesses the extent to which respondents display cultural awareness and sensitivity. The subscale assessing behaviour, in contrast, focuses on actions and attitudes that demonstrate cultural competence in practice. Over time, the questionnaire has been subject to a number of revisions. The initial draft consisted of 45 items, whereas later versions comprised 25 items (Schim et al., 2003) and 27 items ((Doorenbos et al., 2005)) In terms of psychometric properties, the study conducted by Doorenbosch et al. (2005) demonstrated satisfactory reliability: full scale a Cronbach's alpha coefficient (α) of 0.92, subscale for awareness and sensitivity α = 0.75 and subscale behaviour α = 0.93. Both the original Cultural Competence Assessment and the psychometrically evaluated version served as the foundation for this study. After careful consideration by the research group, three items from the evaluated version, which are not in the original Cultural Competence Assessment, were incorporated into the initial version of our study: *'I enjoy working with people who are culturally different from me', 'I welcome feedback from co-workers about how I relate to others with different cultures'*, and *'I learn from co-workers about people with different cultural heritages'*. This process aligns with the cross-cultural adaptation guidelines set forth by Epstein et al. (2015), which recognises the necessity of refining instruments in order to maintain their relevance and applicability in new cultural contexts. Accordingly, the Cultural Competence Assessment scale used in this study comprised 29 items, with a 5-point Likert scale. Respondents could receive scores ranging from 1 point (strongly disagree) to 5 points (strongly agree) per question. As the questionnaire contains both positively and negatively worded items, the negatives were recoded to ensure that higher scores always indicate higher cultural competence. The total score for cultural competence was determined by summing the individual item scores. The potential score range was from 29 to 145, with higher scores indicating higher cultural competence.

### 2.2 Design and participants

We used a cross-sectional design and conducted the study in two phases:

The first phase focused on the translation and cross-cultural adaptation of the English Cultural Competence Assessment into Dutch. This phase aimed to ensure that the Dutch version of the scale captured the intended meaning after translation.

The second phase involved the assessment of the construct validity and reliability of the Dutch version of the Cultural Competence Assessment. This phase aimed to evaluate how well the Dutch questionnaire reflected the cultural competence among nurses.

We invited nurses with a master's degree or qualifications at levels 3, 4, or 6 on the European Qualifications Framework, employed in healthcare organisations in the Netherlands, to participate in the study. Nurses holding doctoral degrees were also eligible for inclusion. Proficiency in the Dutch language was a requirement for participation, reflecting the linguistic context of the Netherlands, where Dutch is the primary language of communication. Healthcare workers other than nurses were excluded. The sample size for the second phase targeted a minimum of 290 nurses, aiming for a ratio of 10 participants per item (Kyriazos, 2018).

The translation, cross-cultural adaptation, and psychometric testing were conducted in accordance with the recommendations set out in the literature (Epstein, Santo and Guillemin, 2015; Streiner, Norman and Cairney, 2015).

### 2.3 Translation fand cross-cultural adaptation

After receiving permission to use the Cultural Competence Assessment from the developers, we conducted the translation process. Firstly, the English Cultural Competence Assessment was translated into Dutch (forward translation) by an independent translation agency certified according to international standards set by the International Organisation for Standardisation. Secondly, the translated version was compared to the original Cultural Competence Assessment by the research team, who engaged in discussions to address any discrepancies in sentences, words, and meanings. The aim was to attain agreement on the translated version. Thirdly, the English translation underwent a thorough examination by two native English speakers. Independently, they evaluated both the Dutch and English versions to ensure linguistic accuracy and cultural appropriateness.

Finally, the research team reviewed the feedback provided by these experts, after which the translated Dutch Cultural Competence Assessment was considered ready for the next phase of the study.

### 2.4 Data collection – psychometric testing

Castor Electronic Data Capture was used for the data collection. Castor Electronic Data Capture is a user-friendly and cloud-based platform designed for collection and management of clinical research data. Before sending out the questionnaire to a large sample of the target population, we assessed the comprehensibility, relevance, acceptability, and feasibility of the Cultural Competence Assessment. The first draft of the translated Dutch Cultural Competence Assessment was evaluated in a sample of 10 nurses. The method for evaluation involved distributing the first version of the Dutch Cultural Competence Assessment to participants via email, granting them access to an online survey in Castor for feedback. Within this survey, both the original English translation and its Dutch counterpart were presented, allowing participants to provide commentary regarding the Cultural Competence Assessment comprehensibility, relevance, acceptability, and feasibility. The participants' insights and suggestions were thoughtfully integrated into the Cultural Competence Assessment, resulting in the development of an updated version.

For the assessment of construct validity and internal consistency, the Cultural Competence Assessment was administered to a larger sample of the target population. The Dutch nurses association, named V&VN, with 105,000 members was requested to place an appeal in its newsletter, where members were invited to participate. Additionally, various healthcare organisations were asked to internally promote the survey, ensuring a broader reach. Data collection took place between January and April 2024.

To gain an insight into the background of the respondents, demographic and professional information was collected. To minimise the occurrence of missing data, all questions were deemed mandatory, except for those related to demographic information. This exception was made in consideration of participants’ privacy concerns.

### 2.5 Data analysis

All statistical analyses were conducted using RStudio (version 4.2.2). Descriptive statistics were used to summarise the demographic and professional characteristics of the participants. These variables were nominal and therefore presented as absolute numbers (n) and percentages (%). The Kaiser-Meyer-Olkin (KMO) measure was used to evaluate the adequacy of the data for factor analysis. Additionally, the Bartlett test was utilised to determine if the correlation matrix significantly deviated from an identity matrix. Moreover, multicollinearity was checked to ensure there were no redundant correlations between the independent variables. Confirmatory Factor Analysis (CFA) was conducted to assess construct validity, using the R package Lavaan, Foreign and SemPlot. Measures of fit included the Root Mean Square Error of Approximation (RMSEA), Standardised Root Mean Square Residual (SRMR), Comparative Fit Index (CFI), and Tucker-Lewis Index (TLI). RMSEA values close to or below 0.06 and SRMR values close to or below 0.08, were indicative of well-fitting models (Hu and Bentler, 1999). CFI and TLI values greater than 0.90 signified a good fit (Hu and Bentler, 1999). Modification indices were used to improve model fit. The internal consistency was evaluated, utilising McDonald's omega as the measure, where McDonald's omega above 0.70 indicated a positive internal consistency (McDonald, 1999).

### 2.6 Ethical considerations

Permission to conduct the study was obtained from the Medical Ethical Committee of Amsterdam University Medical Centre (ref. no. 2023.0819). Data management was executed in compliance with the European General Data Protection Regulation and the research code of Amsterdam Medical Centre. Data from surveys were coded to maintain anonymity and confidentiality of all participants. Participants’ privacy was protected by using anonymised data for reporting and analysis. Access to the original source data was limited to designated members of the research team (PS, JM, and AE). The data collected during the study will be retained for 15 years. To ensure data security, all data and research documentation will be stored on secure servers of Amsterdam Medical Centre.

## 3 Results

### 3.1 Translation and cross-cultural adaptation

After forward translation and comparison of the translated Cultural Competence Assessment with the original Cultural Competence Assessment, two items were removed because they were found to be highly-similar in content to other items in the scale, leading to redundancy: “*Aspects of cultural diversity need to be assessed for each individual, group, and organization*” and “*I document the adaptations I make with clients if I provide direct client services*.” Due to difficulties in understanding words and to ensure understandability in the Dutch context, several textual changes were made: “*cultural heritage*” was changed into “*cultural background*” (item 9, awareness and sensitivity, and item 3, behaviour). “*Families, groups and organizations*,” became “*relatives*” (item 11, awareness and sensitivity). “*Resource books and other materials*” was changed into “*sources*” (item 4, behaviour). “*Explanations of health and illness*,” was changed into “*ideas about health and illness*” (item 5, behaviour). “*Generalizations*” was changed into “*preconception*” (item 7, behaviour) and finally “*cultural assessment*” was changed into “*cultural preferences*” (item 13, behaviour). The translated Cultural Competence Assessment was critically reviewed by two independent English native speakers. As a result of this evaluation, further refinements were made to the text. The word “*race*” was changed into “*descent*” (item 2, awareness and sensitivity), and the word “*explanations*” was changed into “*views*”.

### 3.2 Psychometric testing

During the pilot testing phase, small textual changes in five statements were made to the Dutch questionnaire to improve comprehension and flow of the questions.

#### 3.2.1 Sample

A total response of 472 was achieved, of which 25 respondents were excluded based on the inclusion and exclusion criteria. This resulted of a sample size of 447 nurses, exceeding the initial target of 270. Demographic and professional data are presented in Table 1.

**Table 1:** Demographic and professional characteristics of the respondents.

	*N*	Frequency	%
Gender	*436*		
Male		51	11.7 %
Female		382	87.6 %
Non-binary		3	0.7 %
Age	*420*		
< 24 years		26	6.2 %
25–34		91	21.7 %
35–44		83	19.8 %
45–54		108	25.7 %
55–64		112	26.7 %
≥65 years		14	3.3 %
Work experience	*422*		
<4 years		42	10.0 %
5–10 years		64	15.2 %
≥11 years		316	74.9 %
Country of birth	*404*		
Netherlands		371	91.8 %
Suriname		4	1.0 %
Belgium		5	1.2 %
Other		20	6.0 %
Religious Affiliation	*380*		
Yes		107	28.2 %
No		273	71.8 %
Highest education	*385*		
< Bachelor's degree		71	18.4 %
≥ Bachelor's degree		314	81.6 %
Current work position	*355*		
Nurse – qualification level 3		6	1.7 %
Registered nurse		134	37.7 %
Specialised nurse		167	47.0 %
Nurse lecturer		4	1.1 %
Nurse practitioner		33	9.3 %
Head nurse/nursing manager		11	3.1 %
Direct patient care	*351*		
Yes		289	82.3 %
No		62	17.7 %

The Bartlett's test gave a significant result (*p* = <0.01), which indicated that the correlation matrix was significantly different from an identity matrix. In addition, an overall Kaiser-Meyer-Olkin value of 0.87 was obtained. This indicated that the data were suitable for factor analysis. Except for one item, the remainder of the individual items had a value greater than 0.7. However, item 2 “*Descent is the most important factor in determining a person's culture*” had a value of 0.55 and was therefore removed from the questionnaire. No evidence of multicollinearity was found.

**Fig. 1:**
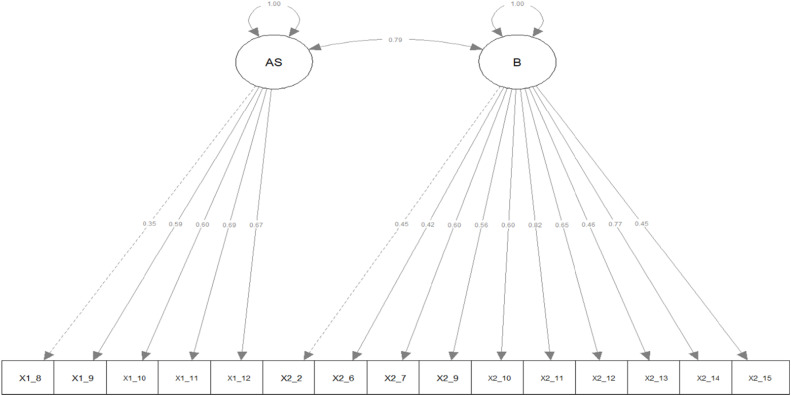
Two factor model.

For the CFA, 26 items were included. The CFA was conducted using both a one-factor model and a two-factor model (given that the English version of the Cultural Competence Assessment consists of two factors: "awareness and sensitivity" and "behaviour" (Doorenbos et al.*,* 2005). The best fit was achieved with a two-factor model consisting of 15 items (Table 2). Five items were deleted from factor one: “awareness and sensitivity,” and seven items were deleted from factor two: “behaviour.” Table 3 gives an overview of the remaining 15 items and their factor loadings. All items loaded significantly on the factors. The factor loadings ranged from 0.35 to 0.77 (Fig. 1). Table 4 provides an overview of the items that were deleted. The McDonald's omega was 0.76, 0.87, and 0.89 of the awareness and sensitivity subscale, behaviour subscale, and the total scale, respectively. This indicated a proficient level of internal consistency.

**Table 2:** Measures of fit CFA.

Factor structure	RMSEA	SRMR	CFI	TLI
One factor (26 items)	0.082	0.074	0.726	0.701
One factor (15 items)	0.072	0.053	0.899	0.881
Two factor (26 items)	0.077	0.072	0.761	0.739
*Two factor (15 items)*	*0.057*	*0.044*	*0.937*	*0.925*
CFA: Confirmatory Factor Analysis; RMSEA: Root Mean Square Error of Approximation; SRMR: Standardised Root Mean Square Residual; CFI: Comparative Fit Index; TLI: Tucker-Lewis Index.

**Table 3:** Final items and factor loadings.

Nr.	Item	Factor Loading
*Awareness and sensitivity*		
1.8	Language barriers are the only difficulties for recent immigrants to the Netherlands.	0.350
1.9	I believe that everyone should be treated with respect no matter what their cultural heritage.	0.587
1.10	I understand that people from different cultures may define the concept of “healthcare” in different ways.	0.601
1.11	I think that knowing about different cultural groups helps direct my work with individuals, families, groups, and organisations.	0.693
1.12	I enjoy working with people who are culturally different from me.	0.674
*Behaviour*		
2.2	I seek information on cultural needs when I identify new people in my work or school.	0.450
2.6	I ask people to tell me about their expectations for health services.	0.419
2.7	I avoid using generalizations to stereotype groups of people.	0.602
2.9	I remove obstacles for people of different cultures when I identify barriers to services	0.563
2.10	I remove obstacles for people of different cultures when people identify barriers to me.	0.602
2.11	I welcome feedback from clients about how I relate to people from different cultures.	0.816
2.12	I find ways to adapt my services to individual and group cultural preferences.	0.648
2.13	I document cultural assessments if I provide direct client services.	0.459
2.14	I welcome feedback from co-workers about how I relate to others with different cultures.	0.770
2.15	I learn from co-workers about people with different cultural heritages.	0.450

**Table 4:** Overview deleted items.

Nr.	Item
Awareness and sensitivity	
1.1	Overall, how competent do you feel working with people who are from cultures different than your own?
1.2	Race is the most important factor in determining a person's culture.
1.3	People with a common cultural background think and act alike.
1.4	Many aspects of culture influence health and healthcare.
1.5	If I know about a person's culture, I don't need to assess their personal preferences for health service.
1.6	Spiritual and religious beliefs are important aspects of many cultural groups.
1.7	Individual people may identify with more than one cultural group.
*Behaviour*	
2.1	I include cultural assessment when I do individual or organisational evaluations.
2.3	I have resource books and other materials available to help me learn about people from different cultures.
2.4	I use a variety of sources to learn about the cultural heritage of other people.
2.5	I ask people to tell me about their own explanations of health and illness.
2.8	I recognize potential barriers to service that might be encountered by different people

## 4 Discussion

We aimed to establish the translation and cross-cultural adaptation and the validity and reliability of the Dutch version of the Cultural Competence Assessment among Dutch nurses. The results of the CFA showed that the translated Cultural Competence Assessment with 15 items and a two-factor model was the most valid and reliable for Dutch nurses. We have extended the findings of previous researchers who evaluated the original Cultural Competence Assessment (Doorenbos et al., 2005). The two-factor structure is in line with the findings of Osmancevic et al. (2022) on the cross-cultural adaptation and validation of the Cultural Competence Assessment in German and with the study by Chae et al. on the cross-cultural adaptation and validation of the Cultural Competence Assessment in Korean (Chae et al., 2018). In our study, we reduced the Cultural Competence Assessment to 15 questions, closely following the structure found in the above-mentioned international validation studies (Chae et al., 2018; Osmancevic et al., 2022). The German and Korean surveys were reduced to 14 and 16 questions, respectively (Chae et al., 2018; Osmancevic et al., 2022). It is noteworthy that 11 questions of the validated Dutch Cultural Competence Assessment corresponded to the validated questions in the German version and nine to those in the Korean version. The discrepancies among these studies can be attributed to cultural differences between the Netherlands, Austria, and Korea.

Previous reviewers have reported on instruments developed to assess cultural competences of healthcare professionals (Gozu et al., 2007; Loftin et al., 2013; Jager et al., 2021). These reviewers have demonstrated that a substantial proportion of instruments lacked methodological strength and validity (Gozu et al., 2007; Jager et al., 2021). However, in addition to the Cultural Competence Assessment, the Cultural Self-Efficacy Scale, the Transcultural Self-Efficacy Tool, and the Inventory for Assessing the Process of Cultural Competence are among the most reliable instruments (Loftin et al., 2013). The Transcultural Self-Efficacy and Cultural Self-Efficacy Scale focus on self-efficacy in specific cultural contexts, while the Inventory for Assessing the Process of Cultural Competence is designed to measure the level of cultural awareness, desire, knowledge, skill, and experience of healthcare providers. It is evident that these instruments are similar to the Cultural Competence Assessment in terms of validity and practical utility in healthcare. However, we preferred the Cultural Competence Assessment because of the subscales behaviour and awareness and sensitivity, both key dimensions of cultural competence relevant to nursing practice. This distinguishes the Cultural Competence Assessment from other instruments.

A number of strengths can be identified in this study. Firstly, during the pilot phase, nurses from various healthcare settings were included, reflecting the sample that was used during psychometric testing of the Cultural Competence Assessment. Secondly, the study sample size of 447 exceeded the initial target of 290. The data were drawn from a heterogeneous sample of nurses from various healthcare settings. This reflected the nursing population in the Netherlands. The overrepresentation of women (88 %) is consistent with the nursing population in the Netherlands, where nursing is dominated by the female gender (CBS, 2023a). The overrepresentation of respondents with a Dutch country of birth (92 %) is also consistent with the nursing population in the Netherlands, where only 6 % of nurses are born abroad (Ministry of Justice, 2021).

We also acknowledge a number of limitations of this study. Firstly, the over-representation of respondents with a bachelor's degree or higher (82 %) is not representative of the nursing profession. In fact, there are 17 % more healthcare workers with a degree at European Qualification Level 4 or lower (CBS, 2024) than nurses with a bachelor's degree or higher. Consequently, the results are partly unrepresentative for the Dutch nursing population and mainly apply to nurses with higher education.

Secondly, the reduction in the number of items in the Dutch version of the Cultural Competence Assessment may lead to concerns regarding the content validity of the instrument. While the revised scale is more concise and easier to administer in clinical settings, there is a possibility that important dimensions of the cultural competence construct were not fully captured. Thirdly, not all demographic questions were mandatory. Consequently, there is uncertainty about the exact working position of the respondents, as only 355 out of 447 respondents answered this question. However, as the distribution of the questionnaire was specifically sent to nurses, it is believed that most respondents were indeed the target population. Fourthly, an analysis of the survey results revealed a ceiling effect. A considerable number of respondents achieved the highest scores. This may have impacted the variability of the data and the sensitivity of the Cultural Competence Assessment. Finally, as the Cultural Competence Assessment is a self-report questionnaire, it is important to consider the limitations of such instruments. The main disadvantage is the risk of social desirability bias, whereby respondents may not answer items truthfully, especially the sensitive questions used in the Cultural Competence Assessment (Demetriou, Ozer and Essau, 2015). This may result in respondents answering in a socially-acceptable way. This phenomenon was considered by explicitly informing participants that there was no right or wrong answer and that this was a self-reflective questionnaire. Other factors thought to be particularly influential in social desirability bias are the presence of others while responding to questions and the respondent's perception of the level of privacy or confidentiality (Brener, Billy and Grady, 2003). By ensuring anonymity and not making demographic questions mandatory, it is likely that the risk of social desirability bias was limited.

While this study focused on nurses, it is important to note that cultural competence is a crucial aspect of healthcare delivery across all professions. All healthcare providers have a responsibility to consider their own cultural competence when providing healthcare to their patients. Therefore, future research is needed to validate the assessment of cultural competence in other healthcare professionals such as doctors and physiotherapists. This may also enhance the credibility of the Cultural Competence Assessment, thereby enhancing its applicability and utility across a broader range of healthcare settings.

Furthermore, additional research on other clinimetric properties, such as measurement error and responsiveness, conducted with particular attention paid to the ceiling effect would be beneficial. Moreover, it would be beneficial for future researchers to collect information on interrelations between cultural competence and socio-demographic variables. It was not possible to assess measurement invariance adequately due to the homogeneity of our group, it is therefore recommended that future researchers should concentrate on exploring the measurement invariance of the Dutch-Cultural Competence Assessment within a sample that is more representative of the Dutch context. Additionally, given that the current sample was primarily nurses with higher education, the application of item response theory could prove beneficial. These steps would serve to enhance the validity, reliability and sensitivity of the Cultural Competence Assessment. As the main focus of our study was to assess the construct validity and reliability, the next step should be to test the comprehensibility, relevance, acceptability and feasibility of the revised Cultural Competence Assessment.

Dutch nurses can use the Cultural Competence Assessment in various ways. Firstly, the Cultural Competence Assessment can assist them in evaluating their own cultural competencies.

Secondly, Dutch nursing instructors can employ the Cultural Competence Assessment in nursing education to assist nursing students in evaluating their cultural competence and utilising the results of the instrument to strengthen their cultural competencies. While nursing students may not yet have the full range of experience that practicing nurses do, the Cultural Competence Assessment can still serve as a useful tool for addressing their understanding of cultural competence and identifying areas for growth. Dutch organisations can use the Cultural Competence Assessment to systematically assess cultural competency gaps within their nursing workforce. Finally, future Dutch researchers can use the Cultural Competence Assessment in future studies where cultural competence is the outcome of interest.

## 5 Conclusion

The dynamic environment of healthcare, with its increasing multicultural diversity, emphasises the need for culturally-sensitive care. With the two-factor, 15-item Dutch Cultural Competence Assessment, we have provided Dutch nurses with an instrument to self-assess their cultural competence, paying special attention to their awareness, sensitivity, and behaviour when caring for people with cultural differences. This contributes to the provision of culturally-sensitive and compassionate care, which consequently improves patient safety and healthcare outcomes in the Netherlands.

## Funding sources

This research did not receive any specific grant from funding agencies in the public, commercial, or not-for-profit sectors.

## CRediT authorship contribution statement

**P.B. Sutharson:** Writing – original draft, Visualization, Validation, Methodology, Investigation, Data curation, Formal analysis, Conceptualization. **J.M. Maaskant:** Writing – review & editing, Supervision, Resources, Conceptualization. **A.M. Eskes:** Writing – review & editing, Supervision, Resources, Conceptualization.

## Declaration of competing interest

The authors declare that they have no known competing financial interests or personal relationships that could have appeared to influence the work reported in this paper.
